# Diagnosis of Primary Trimethylaminuria in an Affected Patient With a Rare Genotype in Sub‐Saharan Africa

**DOI:** 10.1002/jmd2.70005

**Published:** 2025-03-12

**Authors:** M. Dercksen, M. Perumal, E. Davoren, D. R. Reed, C. Murry‐Maritz, R. van der Sluis, S. Mason

**Affiliations:** ^1^ Centre for Human Metabolomics, North‐West University Potchefstroom South Africa; ^2^ Monell Chemical Senses Center Philadelphia USA; ^3^ Private Practice South Africa; ^4^ Biomedical and Molecular Metabolism Research, Faculty of Natural and Agricultural Sciences, North‐West University Potchefstroom South Africa

## Abstract

Primary trimethylaminuria (TMAU) is characterized by systemic accumulation of trimethylamine (TMA) due to the deficient activity of flavin‐containing monooxygenase 3 (FMO3). The disorder does not have detrimental pathophysiological consequences, but patients develop psychological symptoms due to the emotionally debilitating bodily odor defined as decaying fish that affects their quality of life. Here, we illustrate the utility of a diagnostic workup on an adolescent with primary TMAU, including biochemical and genetic investigations that confirm the diagnosis. A direct substrate (TMA) loading protocol was used, followed by the collection of urine samples at predetermined intervals. The conversion of TMA to trimethylamine oxide (TMAO), monitored by ^1^H‐NMR spectrometry, showed a compromised FMO3 metabolic capacity at baseline, becoming more pronounced after loading commenced. The eight coding exons of the *FMO3* gene were Sanger sequenced, revealing a homozygous missense variant, c.23T>C (p.Ile8Thr), as well as two known homozygous variants, c.472G>A (p.Glu158Lys) and c.923A>G (pGlu308Gly), associated with no to mild presentation of TMAU. The advantage of direct substrate‐to‐product monitoring is the elimination of alternative contributors to the odor that would result in the diagnosis of secondary TMAU. The combined functional and genetic approach provided adequate evidence to describe the first primary TMAU patient reported in sub‐Saharan Africa with a genotype not yet described in a homozygous state. Our findings motivate a comprehensive biochemical and genetic approach to discriminate between primary and secondary TMAU. Subsequently, this targeted approach can provide advice on therapeutic management for optimal emotional well‐being.

1

**Summary**

- The study described the diagnosis, via biochemical‐ and genetic testing, of the first patient in Sub‐Saharan Africa affected by primary trimethylaminuria (TMAU).

## 2 Introduction

Flavin‐containing monooxygenase 3 (FMO3) deficiency (OMIM: 136132) causes primary trimethylaminuria (TMAU) (OMIM: 602079), also known as Fish Odor Disease. Humbert et al. [1], characterized the condition, showing that body odor, resembling that of decaying fish, is due to the excretion of the accumulating trimethylamine (TMA) in breath, saliva, sweat, urine, and reproductive fluids of TMAU patients [1, 2]. Although no physical symptoms are observed in this condition, various reports have indicated psychological consequences including depression and suicidal tendencies [2]. A recent UK survey in a TMAU cohort of 44 patients indicated ostracism in either the workplace (90%) or social settings (88%) as the primary complaint by patients [3].

FMO3 is responsible for the oxidation of TMA to trimethylamine‐N‐oxide (TMAO) and is localized in choline metabolism, as depicted by Mason and Dercksen 2024 [4]. Dietary choline is converted to betaine in the gut followed by biochemical conversion to TMA and oxidation to TMAO. The formation from choline to betaine to TMA is presumed to be via the gut microbiome [4]. Biallelic pathogenic variants in the *FMO3* gene result in primary TMAU [5, 6, 7]. while secondary/acquired TMAU is not directly related to genetic etiology [7, 8]. The latter may occur (i) after a viral infection; (ii) due to enzyme immaturity in early childhood which resolves with time; (iii) with hormonal changes; and/or (iv) with precursor overload [7, 8]. Liver disease and gut dysbiosis, including *Helicobacter pylori* infection, have also been associated with secondary TMAU [8, 9]. Some studies showed that secondary TMAU may be associated with missense variants p.Glu158Gly and p.Glu308Gly [9, 10]. Various population groups, including British, Asian, and African American, have been described with mild to severe TMAU [7, 11, 12]. An estimated incidence of heterozygous carriers for severe/chronic primary TMAU is 0.5%–1% [13]. The well‐established database, https://databases.lovd.nl/shared/genes/FMO3 (Updated on February 26, 2024), contains pathogenic, common, and benign variants, and illustrates the influence on FMO3 expression.

A TMA precursor oral loading test with subsequent measurement of TMA and TMAO excretion, followed by the calculation of the FMO3 oxidation capacity in percentage (TMA/(TMA + TMAO) multiplied by 100) over a specific period is recommended for the biochemical diagnosis of TMAU [2, 7]. Murphey et al. 2000 emphasized that a loading protocol is advantageous in distinguishing between TMAU carriers or affected patients with a causative genotype [14]. Three loading protocols have been described in the literature, namely: (i) the Nijmegen protocol—eating a marine fish meal (300 g) with sampling at 2‐ to 12‐h postloading [15]; (ii) a choline loading with sampling done at various time intervals specific to the described laboratory protocols [16, 17, 18, 19, 20]; or (iii) direct loading with TMA with sampling before and after, for 4–6 h, which was initially described by Al‐Waiz et al. 1989 [16] and supported by the national human genome research institute (https://www.genome.gov/Genetic‐Disorders/Trimethylaminuria).

The aim of this study was to use the direct TMA loading protocol, and subsequent dual biochemical (on ^1^H‐NMR spectrometry) [4, 14] and genetic diagnostic approach [10] to diagnose a severely affected patient with TMAU with a distinct genotype, not yet described in the literature.

## 3 Materials and Methods

### 3.1 Preparation of TMA Loading Mixture

The TMA loading mixture was prepared by adding 1.3 mL of TMA solution (43%–49%) to 250 mL of orange juice and thoroughly mixed. In our experience, this masks the odor of the TMA. The TMA–orange juice mixture was frozen and couriered, on dry ice, to the referring pathology laboratory.

### 3.2 TMA Loading and Sample Collection

A urine sample was collected before the loading test, which served as the baseline (time 0). The TMA mixture was then administered orally. Subsequently, urine samples were collected every hour for the next 4 h and clearly labeled on the containers. The procedure was performed under medical supervision, and a questionnaire was completed by the patient. To maintain the cold chain, samples were frozen immediately after each collection. The protocol was not performed near or during the patient's menstrual period. Ethylenediaminetetraacetic acid (EDTA) blood was collected for DNA extraction at any time interval. Unfortunately, the blood samples of the parents were not available for genetic testing.

### 3.3 Sample Preparation and ^1^H‐NMR Analysis

^1^H‐NMR analysis of the urine samples was performed as described by the protocol of Mason and Dercksen (2024) [4]. The latter source also illustrates the control (unaffected) versus TMAU‐affected NMR spectra. Established reference ranges are summarized in Table 1.

**TABLE 1:** TMA loading results.

Time	TMA (mmol/mol creatinine)	TMAO (mmol/mol creatinine)	FMO3 metabolic capacity^b^ in %
Baseline	39.36^a^	23.23	37.1
Time 1	854.45	24.96	2.8
Time 2	1607.15	29.17	1.8
Time 3	2144.85	35.01	1.7
Time 4	1901.02	47.86	2.5

^a^ Baseline ref.: < 20 mmol/mol creatinine [2].

^b^ FMO3 metabolic capacity: < 43% for severe cases; 44%–70% for moderate cases; 71%–92% for mild cases; > 92% for unaffected individuals [2, 7]. Wildtype/p.Glu158Lys and p.Glu308Gly may have some effect on the FMO_3_ metabolic capacity but typically within the normal reference range (90%–100%) [2, 7, 18, 19].

### 3.4 PCR Amplification and Sanger Sequencing of the Coding Exons of *FMO3*

Human genomic DNA was isolated from whole blood with the NucleoSpin kit supplied by Machery‐Nagel. PCR analysis on genomic DNA using primers specific for FMO3 protein‐coding Exons 2 through Exon 9 was performed following previously described methods [10]. PCR products were sequenced at the Monell Chemical Senses Centre, Pennsylvania in Philadelphia, PA. Sequence chromatograms were analyzed using FinchTV v.1.4.0 (Geospiza; Seattle, WA) (Geospiza Inc.; Seattle, WA, USA; http://www.geospiza.com) and alignments to the *FMO3* reference sequence (NG_012690.1) were done using MEGAX [21].

## 4 Results

### 4.1 Clinical History of the Index Patient

An 11‐year‐old Caucasian female was referred to our unit for TMAU biochemical and genetic investigations by the consulting dietician. She, the only child of nonconsanguineous parents, described her symptoms to the dietician as a “fish‐like” bodily odor with no other chronic conditions mentioned. She was subjected to teasing in school due to an intermittent (around the time of her menses) foul odor, and the school was concerned about her personal hygiene. The family reported that the fish odor was most prominent when the child consumed seafood. No information before the age of 11 years was provided by the clinical team.

A questionnaire, which accompanied the information pack on the TMAU loading protocol, was completed by the patient (Refer to Data S3). Interestingly, the patient did not report a significant difference in odor pre and postloading. In contrast, when the samples did arrive at our laboratory for testing, the fishy smell was extreme. This might indicate that the patient forms part of the 7% of the human population who are anosmic to the odor of TMA [22].

After receiving the diagnosis of primary TMAU, the patient has followed a strict diet according to recommendations made by the National Human Genome Research Institute (NHGRI) (https://www.genome.gov/Genetic‐Disorders/Trimethylaminuria). The diet entailed the limitation of choline (including fish) intake. Initially, after consultation with a geneticist, activated charcoal was taken twice daily for 10 days, accompanied by copper chlorophyllin taken three times daily after meals for 3 weeks. The clinician prescribed antibiotics (not specified in their report) twice annually to maintain a healthy microbiome. In addition, soaps with a moderate pH (5.5–5.6) were recommended. By maintaining a neutral skin pH, less volatile TMA (pH 9.8) secretion was experienced. Cofactor supplementation, namely Vitamin B2 (riboflavin) was additionally recommended to promote any residual FMO3 activity. The dosage of mentioned supplementation is defined on the NHGRI website. The management of her condition has improved considerably as she tries to limit triggers associated with TMAU. She is currently a functioning young adult (19 years) making a valued contribution to society.

### 4.2 Biochemical Assessment Before and After the TMA Loading Test

**FIGURE 1:**
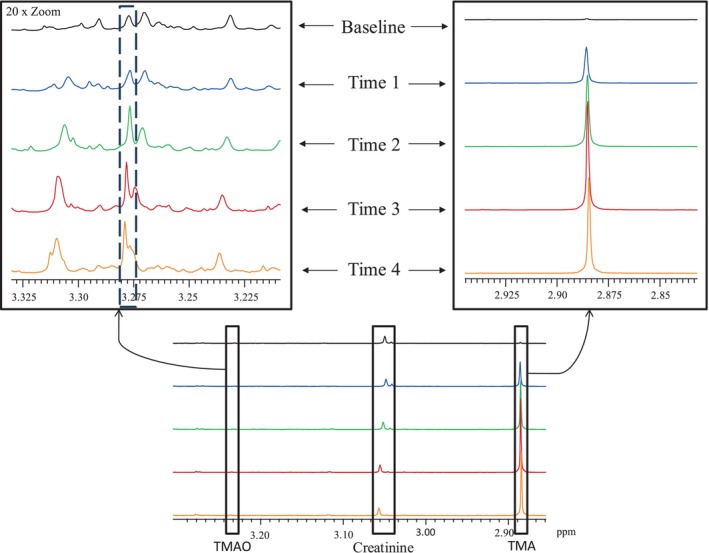
Visual representation of the ^1^H‐NMR results of TMA and TMAO during the loading test. TMA is present at 2.88 ppm and TMAO is present at 3.278 ppm.

The FMO3 metabolic capacity before loading was less than 43%, indicative of a severe case of TMAU. The postload urine collected over a 4‐h loading showed a clear deficiency in the oxidation of TMA to TMAO, with an average FMO3 metabolic capacity of 1.7%–2.8% (Table 1 and Figure 1).

### 4.3 PCR Amplification and Sanger Sequencing of the Coding Exons of *FMO3*

To identify *FMO3* variants within the coding region of the *FMO3* gene, exons 2–9 were PCR amplified and sent for Sanger sequencing. The resulting exon sequences were aligned with the *FMO3* reference sequence (NM_001002294.3). Three homozygous missense variants were identified (Table 2).

**TABLE 2:** *FMO3* missense variants.

SNP	Location	Exon	REF	ALT	REF codon	ALT codon	MAF	Amino acid change	Patient genotype	Chromatogram
rs565935391	1:171092681	2	T	C	ATT	ACT	0.01	Ile8Thr	C/C	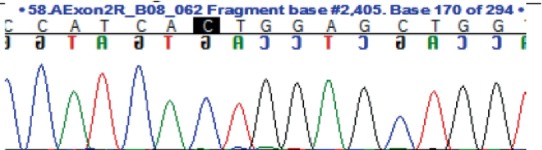
rs2266782	1:171107825	4	G	A	GAG	AAG	0.5	Glu158Lys	A/A	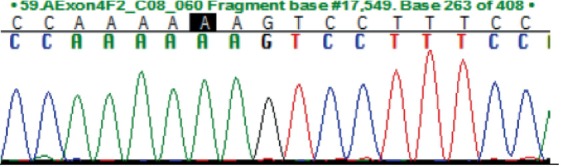
rs2266780	1:171114102	7	A	G	GAG	GGG	0.24	Glu308Gly	G/G	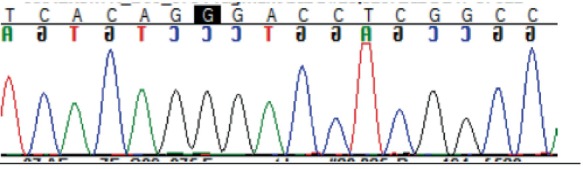

*Note:* Ensembl database accessed August 2, 2024. For the full‐length nucleic acid and amino acid sequence see File S1 For the full‐length nucleic acid and amino acid sequence see File S2.

Abbreviations: ALT codon, alternative codon; ALT, alternative allele; Exon, exon number; Location, base pair position relative to map assembly GRCh38.p14; MAF, highest population minor allele frequency; REF codon, reference codon; REF, reference allele; SNP, single nucleotide polymorphism.

## 5 Discussion

The direct TMA loading, subsequent biochemical monitoring (before and after TMA loading), and genetic testing differentiated primary TMAU (due to *FMO3* mutations) from secondary TMAU [11, 15]. Our method revealed the true metabolic FMO3 capacity and eliminated any secondary contributors (other than TMA) to the odor. The *FMO3* Sanger sequencing confirmed the diagnosis.

This is the first report associating the p.Ile8Thr missense variant with severe TMAU. It is currently classified as a variant of uncertain significance in ClinVar (https://www.ncbi.nlm.nih.gov/clinvar/) and Franklin by Genoox (https://franklin.genoox.com). The variant has, however, been reported on Ensembl (https://www.ensembl.org/index.html) in the general population, but at an extremely low frequency. The Sift [23] and Polyphen [24] scores for this variant predict that it might be a deleterious variant. This is supported by the exceptionally low frequency of the C allele in the gnomAD exomes r2.1.1 database, where it has been reported in two heterozygote individuals (a total of 125 748 exomes; one from the non‐Finnish European population and one population not assigned). Additionally, the common missense variants p.Glu158Lys and p.Glu308Gly [9, 18] were also identified in homozygous state. Some studies have indicated that cis‐linkage of p.Glu158Lys and p.Glu308Gly does show a decrease in the FMO3 activity to a variable extent [18, 19]. The variation and its effect are highly dependent on population groups, and this linkage is more prominent in Caucasian European and American groups and significantly lower in the African (West and East) groups, except for the San population—a sub‐Saharan African community [19]. Furthermore, rare disease Caucasian patients in South Africa, with unique genotypes, primarily consist of European descendants who colonized South Africa in the 17th and 18th centuries [25]. The TMAU diagnostic protocol described in this paper has been applied within our laboratory for over 8 years [25]. Secondary TMAU (based on FMO3 capacity results which were essentially normal) associated with common variants such as p.Glu158Gly and/or to a lesser extent p.Glu308Gly have been observed in the South African population groups (Caucasian, Black, mixed‐race, and Indian).

## 6 Conclusion

The severity of the clinical features, biochemical findings, and mutational prediction tools illustrate that the described genotype results in primary TMAU. The patient is currently doing well, and her symptoms are moderately controlled by the dietary elimination of seafood. Treatment strategies, as described by Schmidt et al. [26] have proven to be effective in improving overall quality of life for this patient. Many potential African patients, part of a TMAU support group, remain untested, and we hope this case report will raise awareness of TMAU testing services in South Africa.

## Author Contributions

We can confirm that all authors took responsibility for the work presented and performed the study in an ethical manner. The collaborators had access to the data and agreed that the paper adds value to the scientific and medical community. No con. M. Dercksen: Biochemical diagnosis of the patient. She provided the outline of the paper and overall contributed to all sections of the paper, more specifically the abstract, results, and conclusion sections. She also presented this research (poster) at the 2018 SSIEM, Greece. M. Perumal: Design of manuscript, compiling the introduction, reference compilation, and checking all grammar and references. E. Davoren: Performed the NMR analysis, provided information on the loading protocol, and contributed to the method section in the manuscript. S. Mason: Documenting the method section of the manuscript including Figure 1 and Table 2. He also did a final grammar check. D.R. Reed: Oversaw the sequencing and reporting of genetic data, contributing to the title of the paper and final outline of the manuscript. R. van der Sluis: Documentation of the genetic sections (methods and results) including the provision of the Supporting Information in the manuscript. She contributed to the introduction and results section. C. Maritz: Referring clinician who initiated diagnostic testing and subsequently offered treatment options to the patient.

## Consent

All procedures followed were in accordance with the ethical standards of the committee responsible for human experimentation (institutional and national) and with the Helsinki Declaration of 1975, as revised in 2000 (5). Consent for publication was obtained from the patient. Ethics approval: This study was conducted under a protocol approved by the Ethics Committee of North‐West University, protocol number NWU‐BB001‐19‐A1 after obtaining informed consent from the patient.

## Conflicts of Interest

The authors declare no Conflicts of Interest.

## Supporting information


Data S1.

Data S2.

Data S3.

## Data Availability

All data are available within the manuscript, including the supplementary documentation.
