# Supplementary material for: Diagnosis of Primary Trimethylaminuria in an Affected Patient With a Rare Genotype in Sub‐Saharan Africa
Source: JIMD Rep. 2025 Mar 12;66(2):e70005. doi: 10.1002/jmd2.70005 (PMC11897904; doi:10.1002/jmd2.70005)
Supplement: Supplementary file 1 — Data S1. [file JMD2-66-e70005-s002.pdf]

Clustal Omega

Multiple Sequence Alignment (MSA)

Welcome to the new **Job Dispatcher** website. We'd love to hear your feedback about the new webpages! ×

Results for Job ID

clustalo-I20241007-122019-0414-45475849-p1m Copy

Resubmission

Tool output

Download

```
CLUSTAL O(1.2.4) multiple sequence alignment


FMO3-201cds:protein_coding        ATGGGGAAGAAAGTGGCCATCATTGGAGCTGGTGTGAGTGGCTTGGCCTCCATCAGGAGC
60
Patient_nucleic_acid_sequence     ATGGGGAAGAAAGTGGCCATCACTGGAGCTGGTGTGAGTGGCTTGGCCTCCATCAGGAGC
60
                                  ********************** *************************************

FMO3-201cds:protein_coding        TGTCTGGAAGAGGGGCTGGAGCCCACCTGCTTTGAGAAGAGCAATGACATTGGGGGCCTG
120
Patient_nucleic_acid_sequence     TGTCTGGAAGAGGGGCTGGAGCCCACCTGCTTTGAGAAGAGCAATGACATTGGGGGCCTG
120
                                  ************************************************************

FMO3-201cds:protein_coding        TGGAAATTTTCAGACCATGCAGAGGAGGGCAGGGCTAGCATTTACAAATCAGTCTTTTCC
180
Patient_nucleic_acid_sequence     TGGAAATTTTCAGACCATGCAGAGGAGGGCAGGGCTAGCATTTACAAATCAGTCTTTTCC
```

```
180
                                   ************************************************************

FMO3-201cds:protein_coding         AACTCTTCCAAAGAGATGATGTGTTTCCCAGACTTCCCATTTCCCGATGACTTCCCCAAC
240
Patient_nucleic_acid_sequence      AACTCTTCCAAAGAGATGATGTGTTTCCCAGACTTCCCATTTCCCGATGACTTCCCCAAC
240
                                   ************************************************************

FMO3-201cds:protein_coding         TTTATGCACAACAGCAAGATCCAGGAATATATCATTGCATTTGCCAAAGAAAAGAACCTC
300
Patient_nucleic_acid_sequence      TTTATGCACAACAGCAAGATCCAGGAATATATCATTGCATTTGCCAAAGAAAAGAACCTC
300
                                   ************************************************************

FMO3-201cds:protein_coding         CTGAAGTACATACAATTTAAGACATTTGTATCCAGTGTAAATAAACATCCTGATTTTGCA
360
Patient_nucleic_acid_sequence      CTGAAGTACATACAATTTAAGACATTTGTATCCAGTGTAAATAAACATCCTGATTTTGCA
360
                                   ************************************************************

FMO3-201cds:protein_coding         ACTACTGGCCAGTGGGATGTTACCACTGAAAGGGATGGTAAAAAAGAATCGGCTGTCTTT
420
Patient_nucleic_acid_sequence      ACTACTGGCCAGTGGGATGTTACCACTGAAAGGGATGGTAAAAAAGAATCGGCTGTCTTT
420
                                   ************************************************************

FMO3-201cds:protein_coding         GATGCTGTAATGGTTTGTTCCGGACATCATGTGTATCCCAACCTACCAAAAGAGTCCTTT
480
Patient_nucleic_acid_sequence      GATGCTGTAATGGTTTGTTCCGGACATCATGTGTATCCCAACCTACCAAAAAAGTCCTTT
480
                                   *************************************************** ********

FMO3-201cds:protein_coding         CCAGGACTAAACCACTTTAAAGGCAAATGCTTCCACAGCAGGGACTATAAAGAACCAGGT
540
Patient_nucleic_acid_sequence      CCAGGACTAAACCACTTTAAAGGCAAATGCTTCCACAGCAGGGACTATAAAGAACCAGGT
540
                                   ************************************************************

FMO3-201cds:protein_coding         GTATTCAATGGAAAGCGTGTCCTGGTGGTTGGCCTGGGGAATTCGGGCTGTGATATTGCC
600
Patient_nucleic_acid_sequence      GTATTCAATGGAAAGCGTGTCCTGGTGGTTGGCCTGGGGAATTCGGGCTGTGATATTGCC
600
                                   ************************************************************

FMO3-201cds:protein_coding         ACAGAACTCAGCCGCACAGCAGAACAGGTCATGATCAGTTCCAGAAGTGGCTCCTGGGTG
660
Patient_nucleic_acid_sequence      ACAGAACTCAGCCGCACAGCAGAACAGGTCATGATCAGTTCCAGAAGTGGCTCCTGGGTG
660
                                   ************************************************************

FMO3-201cds:protein_coding         ATGAGCCGGGTCTGGGACAATGGTTATCCTTGGGACATGCTGCTCGTCACTCGATTTGGA
720
Patient_nucleic_acid_sequence      ATGAGCCGGGTCTGGGACAATGGTTATCCTTGGGACATGCTGCTCGTCACTCGATTTGGA
720
                                   ************************************************************

FMO3-201cds:protein_coding         ACCTTCCTCAAGAACAATTTACCGACAGCCATCTCTGACTGGTTGTACGTGAAGCAGATG
780
Patient_nucleic_acid_sequence      ACCTTCCTCAAGAACAATTTACCGACAGCCATCTCTGACTGGTTGTACGTGAAGCAGATG
780
```

```
                                  ************************************************************

FMO3-201cds:protein_coding        AATGCAAGATTCAAGCATGAAAACTATGGCTTGATGCCTTTAAATGGAGTCCTGAGGAAA
840
Patient_nucleic_acid_sequence     AATGCAAGATTCAAGCATGAAAACTATGGCTTGATGCCTTTAAATGGAGTCCTGAGGAAA
840
                                  ************************************************************

FMO3-201cds:protein_coding        GAGCCTGTATTTAACGATGAGCTCCCAGCAAGCATTCTGTGTGGCATTGTGTCCGTAAAG
900
Patient_nucleic_acid_sequence     GAGCCTGTATTTAATGATGAGCTCCCAGCAAGCATTCTGTGTGGCATTGTGTCCGTAAAG
900
                                  ************** *********************************************

FMO3-201cds:protein_coding        CCTAACGTGAAGGAATTCACAGAGACCTCGGCCATTTTTGAGGATGGGACCATATTTGAG
960
Patient_nucleic_acid_sequence     CCTAACGTGAAGGAATTCACAGGGACCTCGGCCATTTTTGAGGATGGGACCATATTTGAG
960
                                  ********************** *************************************

FMO3-201cds:protein_coding        GGCATTGACTGTGTAATCTTTGCAACAGGGTATAGTTTTGCCTACCCCTTCCTTGATGAG
1020
Patient_nucleic_acid_sequence     GGCATTGACTGTGTAATCTTTGCAACAGGGTATAGTTTTGCCTACCCCTTCCTTGATGAG
1020
                                  ************************************************************

FMO3-201cds:protein_coding        TCTATCATCAAAAGCAGAAACAATGAGATCATTTTATTTAAAGGAGTATTTCCTCCTCTA
1080
Patient_nucleic_acid_sequence     TCTATCATCAAAAGCAGAAACAATGAGATCATTTTATTTAAAGGAGTATTTCCTCCTCTA
1080
                                  ************************************************************

FMO3-201cds:protein_coding        CTTGAGAAGTCAACCATAGCAGTGATTGGCTTTGTCCAGTCCCTTGGGGCTGCCATTCCC
1140
Patient_nucleic_acid_sequence     CTTGAGAAGTCAACCATAGCAGTGATTGGCTTTGTCCAGTCCCTTGGGGCTGCCATTCCC
1140
                                  ************************************************************

FMO3-201cds:protein_coding        ACAGTTGACCTCCAGTCCCGCTGGGCAGCACAAGTAATAAAGGGAACTTGTACTTTGCCT
1200
Patient_nucleic_acid_sequence     ACAGTTGACCTCCAGTCCCGCTGGGCAGCACAAGTAATAAAGGGAACTTGTACTTTGCCT
1200
                                  ************************************************************

FMO3-201cds:protein_coding        TCTATGGAAGACATGATGAATGATATTAATGAGAAAATGGAGAAAAAGCGCAAATGGTTT
1260
Patient_nucleic_acid_sequence     TCTATGGAAGACATGATGAATGATATTAATGAGAAAATGGAGAAAAAGCGCAAATGGTTT
1260
                                  ************************************************************

FMO3-201cds:protein_coding        GGCAAAAGCGAGACCATACAGACAGATTACATTGTTTATATGGATGAACTCTCCTCCTTC
1320
Patient_nucleic_acid_sequence     GGCAAAAGCGAGACCATACAGACAGATTACATTGTTTATATGGATGAACTCTCCTCCTTC
1320
                                  ************************************************************

FMO3-201cds:protein_coding        ATTGGGGCAAAGCCCAACATCCCATGGCTGTTTCTCACAGATCCCAAATTGGCCATGGAA
1380
Patient_nucleic_acid_sequence     ATTGGGGCAAAGCCCAACATCCCATGGCTGTTTCTCACAGATCCCAAATTGGCCATGGAA
1380
                                  ************************************************************
```

```
FMO3-201cds:protein_coding          GTTTATTTTGGCCCTTGTAGTCCCTACCAGTTTAGGCTGGTGGGCCCAGGGCAGTGGCCA
1440
Patient_nucleic_acid_sequence       GTTTATTTTGGCCCTTGTAGTCCCTACCAGTTTAGGCTGGTGGGCCCAGGGCAGTGGCCA
1440
                                    ************************************************************

FMO3-201cds:protein_coding          GGAGCCAGAAATGCCATACTGACCCAGTGGGACCGGTCGTTGAAACCCATGCAGACACGA
1500
Patient_nucleic_acid_sequence       GGAGCCAGAAATGCCATACTGACCCAGTGGGACCGGTCGTTGAAACCCATGCAGACACGA
1500
                                    ************************************************************

FMO3-201cds:protein_coding          GTGGTCGGGAGACTTCAGAAGCCTTGCTTCTTTTTCCATTGGCTGAAGCTCTTTGCAATT
1560
Patient_nucleic_acid_sequence       GTGGTCGGGAGACTTCAGAAGCCTTGCTTCTTTTTCCATTGGCTGAAGCTCTTTGCAATT
1560
                                    ************************************************************

FMO3-201cds:protein_coding          CCTATTCTGTTAATCGCTGTTTTCCTTGTGTTGACCTAA      1599
Patient_nucleic_acid_sequence       CCTATTCTGTTAATCGCTGTTTTCCTTGTGTTGACCTAA      1599
                                    ***************************************
```

Alignment with colours

Show

If you use this service, please consider citing the following publication: The EMBL-EBI Job Dispatcher sequence analysis tools framework in 2024. More information about this bioinformatics application can be found in its bio.tools record.

Please read the provided Help & Privacy before seeking help from our support staff. If you have any feedback or experienced any issues please let us know via EMBL-EBI Support. Read our Privacy Notice if you are concerned with your privacy and how we handle personal information.

EMBL-EBI, Wellcome Genome Campus, Hinxton, Cambridgeshire, CB10 1SD, UK. Tel: +44 (0)1223 49 44 44 Full contact details

Copyright © EMBL 2024 EMBL-EBI is part of the European Molecular Biology Laboratory Terms of use
