# Supplementary material for: Diagnosis of Primary Trimethylaminuria in an Affected Patient With a Rare Genotype in Sub‐Saharan Africa
Source: JIMD Rep. 2025 Mar 12;66(2):e70005. doi: 10.1002/jmd2.70005 (PMC11897904; doi:10.1002/jmd2.70005)
Supplement: Supplementary file 2 — Data S2. [file JMD2-66-e70005-s001.pdf]

Clustal Omega

Multiple Sequence Alignment (MSA)

Welcome to the new **Job Dispatcher** website. We'd love to hear your feedback about the new webpages! ×

Results for Job ID

clustalo-I20241007-122922-0259-10311332-p1m Copy

Resubmission

Tool output

Download

```
CLUSTAL O(1.2.4) multiple sequence alignment


FMO3-201        MGKKVAIIGAGVSGLASIRSCLEEGLEPTCFEKSNDIGGLWKFSDHAEEGRASIYKSVFS  60
FMO3_patient    MGKKVAITGAGVSGLASIRSCLEEGLEPTCFEKSNDIGGLWKFSDHAEEGRASIYKSVFS  60
                ******* ****************************************************

FMO3-201        NSSKEMMCFPDFPFPDDFPNFMHNSKIQEYIIAFAKEKNLLKYIQFKTFVSSVNKHPDFA  120
FMO3_patient    NSSKEMMCFPDFPFPDDFPNFMHNSKIQEYIIAFAKEKNLLKYIQFKTFVSSVNKHPDFA  120
                ************************************************************

FMO3-201        TTGQWDVTTERDGKKESAVFDAVMVCSGHHVYPNLPKESFPGLNHFKGKCFHSRDYKEPG  180
FMO3_patient    TTGQWDVTTERDGKKESAVFDAVMVCSGHHVYPNLPKKSFPGLNHFKGKCFHSRDYKEPG  180
                *************************************:**********************

FMO3-201        VFNGKRVLVVGLGNSGCDIATELSRTAEQVMISSRSGSWVMSRVWDNGYPWDMLLVTRFG  240
FMO3_patient    VFNGKRVLVVGLGNSGCDIATELSRTAEQVMISSRSGSWVMSRVWDNGYPWDMLLVTRFG  240
                ************************************************************
```

```
FMO3-201        TFLKNNLPTAISDWLYVKQMNARFKHENYGLMPLNGVLRKEPVFNDELPASILCGIVSVK  300
FMO3_patient    TFLKNNLPTAISDWLYVKQMNARFKHENYGLMPLNGVLRKEPVFNDELPASILCGIVSVK  300
                ************************************************************

FMO3-201        PNVKEFTETSAIFEDGTIFEGIDCVIFATGYSFAYPFLDESIIKSRNNEIILFKGVFPPL  360
FMO3_patient    PNVKEFTGTSAIFEDGTIFEGIDCVIFATGYSFAYPFLDESIIKSRNNEIILFKGVFPPL  360
                ******* ****************************************************

FMO3-201        LEKSTIAVIGFVQSLGAAIPTVDLQSRWAAQVIKGTCTLPSMEDMMNDINEKMEKKRKWF  420
FMO3_patient    LEKSTIAVIGFVQSLGAAIPTVDLQSRWAAQVIKGTCTLPSMEDMMNDINEKMEKKRKWF  420
                ************************************************************

FMO3-201        GKSETIQTDYIVYMDELSSFIGAKPNIPWLFLTDPKLAMEVYFGPCSPYQFRLVGPGQWP  480
FMO3_patient    GKSETIQTDYIVYMDELSSFIGAKPNIPWLFLTDPKLAMEVYFGPCSPYQFRLVGPGQWP  480
                ************************************************************

FMO3-201        GARNAILTQWDRSLKPMQTRVVGRLQKPCFFFHWLKLFAIPILLIAVFLVLT  532
FMO3_patient    GARNAILTQWDRSLKPMQTRVVGRLQKPCFFFHWLKLFAIPILLIAVFLVLT  532
                ****************************************************
```

Alignment with colours

Show

If you use this service, please consider citing the following publication: The EMBL-EBI Job Dispatcher sequence analysis tools framework in 2024. More information about this bioinformatics application can be found in its bio.tools record.

Please read the provided Help & Privacy before seeking help from our support staff. If you have any feedback or experienced any issues please let us know via EMBL-EBI Support. Read our Privacy Notice if you are concerned with your privacy and how we handle personal information.

EMBL-EBI is the home for big data in biology.

We help scientists exploit complex information to make discoveries that benefit humankind.

SERVICES

Data resources and tools
Data submission
Support and feedback
Licensing
Long-term data preservation

RESEARCH

Publications
Research groups
Postdocs and PhDs

EMBL-EBI, Wellcome Genome Campus, Hinxton, Cambridgeshire, CB10 1SD, UK. Tel: +44 (0)1223 49 44 44 Full contact details

Copyright © EMBL 2024 EMBL-EBI is part of the European Molecular Biology Laboratory Terms of use
