# Supplementary material for: Diagnosis of Primary Trimethylaminuria in an Affected Patient With a Rare Genotype in Sub‐Saharan Africa
Source: JIMD Rep. 2025 Mar 12;66(2):e70005. doi: 10.1002/jmd2.70005 (PMC11897904; doi:10.1002/jmd2.70005)
Supplement: Supplementary file 3 — Data S3. [file JMD2-66-e70005-s003.pdf]

PERSONAL ASSESMENTQUESTIONNAIRE BEFORE AND DURING TMAU LOADING:

**Name and Surname:**	**Index patient***		
**Date of birth:**	**11-years***		
**Gender (male/female):**	**Female**		
**Diet: 24 hours leading up to TMAU loading:**	**Toast with chocolate spread**<br>**Roasted chicken thigh with vegetables (mixed)**		
**Medication intake (including dosage) prior and during TMA loading**	**No medication**		
**Sample information**	**Time of urine collection**	**Rate odour observed (0 being no odour to 5 being extreme odor) #**	**Description of odour (3 words) #**
**Time 0 (before loading)**	10:40	1	Normal urine smell.
**Time 1**	11:40	1	Normal urine smell.
**Time 2**	12:40	2	Urine has a strong “salty” smell (light in color), skin smells fishy.
**Time 3**	13:40	0-1	Urine smelled like nothing. Skin smells fishy.
**Time 4**	14:40	0-1	Urine smelled like nothing, Skin smells fishy.

*Details were not disclosed to protect the identity of the patient.
#The odor rating and description was the patient’s perspective as documented by the mother at time of collection.
